# Processing of *Candida albicans* Ece1p Is Critical for Candidalysin Maturation and Fungal Virulence

**DOI:** 10.1128/mBio.02178-17

**Published:** 2018-01-23

**Authors:** Jonathan P. Richardson, Selene Mogavero, David L. Moyes, Mariana Blagojevic, Thomas Krüger, Akash H. Verma, Bianca M. Coleman, Jacinto De La Cruz Diaz, Daniela Schulz, Nicole O. Ponde, Giulia Carrano, Olaf Kniemeyer, Duncan Wilson, Oliver Bader, Simona I. Enoiu, Jemima Ho, Nessim Kichik, Sarah L. Gaffen, Bernhard Hube, Julian R. Naglik

**Affiliations:** a Mucosal and Salivary Biology Division, Dental Institute, King’s College London, London, United Kingdom; b Department of Microbial Pathogenicity Mechanisms, Leibniz Institute for Natural Product Research and Infection Biology, Hans Knoell Institute (HKI), Jena, Germany; c Centre for Host-Microbiome Interactions, Mucosal and Salivary Biology Division, Dental Institute, King’s College London, London, United Kingdom; d Department of Molecular and Applied Microbiology, Leibniz Institute for Natural Product Research and Infection Biology, Hans Knoell Institute (HKI), Jena, Germany; e Division of Rheumatology and Clinical Immunology, University of Pittsburgh, Pittsburgh, Pennsylvania, USA; f Medical Research Council Centre for Medical Mycology at the University of Aberdeen, Aberdeen Fungal Group, Institute of Medical Sciences, Foresterhill, Aberdeen, United Kingdom; g Institute for Medical Microbiology, University Medical Center Göttingen, Göttingen, Germany; h Friedrich Schiller University, Jena, Germany; Universidade de Sao Paulo

**Keywords:** *Candida albicans*, candidalysin, fungal infection, kexin, mucosal immunity

## Abstract

*Candida albicans* is an opportunistic fungal pathogen responsible for superficial and life-threatening infections in humans. During mucosal infection, *C. albicans* undergoes a morphological transition from yeast to invasive filamentous hyphae that secrete candidalysin, a 31-amino-acid peptide toxin required for virulence. Candidalysin damages epithelial cell plasma membranes and stimulates the activating protein 1 (AP-1) transcription factor c-Fos (via p38–mitogen-activated protein kinase [MAPK]), and the MAPK phosphatase MKP1 (via extracellular signal-regulated kinases 1 and 2 [ERK1/2]–MAPK), which trigger and regulate proinflammatory cytokine responses, respectively. The candidalysin toxin resides as a discrete cryptic sequence within a larger 271-amino-acid parental preproprotein, Ece1p. Here, we demonstrate that kexin-like proteinases, but not secreted aspartyl proteinases, initiate a two-step posttranslational processing of Ece1p to produce candidalysin. Kex2p-mediated proteolysis of Ece1p after Arg61 and Arg93, but not after other processing sites within Ece1p, is required to generate immature candidalysin from Ece1p, followed by Kex1p-mediated removal of a carboxyl arginine residue to generate mature candidalysin. *C. albicans* strains harboring mutations of Arg61 and/or Arg93 did not secrete candidalysin, were unable to induce epithelial damage and inflammatory responses *in vitro*, and showed attenuated virulence *in vivo* in a murine model of oropharyngeal candidiasis. These observations identify enzymatic processing of *C. albicans* Ece1p by kexin-like proteinases as crucial steps required for candidalysin production and fungal pathogenicity.

## IMPORTANCE

*Candida albicans* is an opportunistic fungal pathogen that causes mucosal infection in millions of individuals worldwide. Successful infection requires the secretion of candidalysin, the first cytolytic peptide toxin identified in any human fungal pathogen. Candidalysin is derived from its parent protein Ece1p. Here, we identify two key amino acids within Ece1p vital for processing and production of candidalysin. Mutations of these residues render *C. albicans* incapable of causing epithelial damage and markedly reduce mucosal infection *in vivo*. Importantly, candidalysin production requires two individual enzymatic events. The first involves processing of Ece1p by Kex2p, yielding immature candidalysin, which is then further processed by Kex1p to produce the mature toxin. These observations identify important steps for *C. albicans* pathogenicity at mucosal surfaces.

## INTRODUCTION

Fungal infections are a major cause of morbidity and mortality in the global population with species of *Candida*, *Cryptococcus*, *Pneumocystis*, and *Aspergillus* contributing to an estimated 2 million life-threatening infections reported each year (1). *Candida albicans* causes both superficial infections at mucosal surfaces (e.g., thrush), which affect millions of people worldwide, and life-threatening bloodstream infections in susceptible individuals (1, 2). *C. albicans* pathogenicity is dependent upon multiple virulence factors, but the yeast-to-hypha morphological transition is recognized as being the most important for mucosal infections. *C. albicans* hypha formation is essential for epithelial damage and immune activation, and it was recently discovered that both processes are driven by the secretion of candidalysin from hyphae (3). Candidalysin is an amphipathic 31-amino-acid (aa) cytolytic peptide toxin that is vitally important for *C. albicans* mucosal infection and functions by destabilizing the integrity of plasma membranes and activating epithelial immunity via the activating protein 1 (AP-1) transcription factor c-Fos (via the p38–mitogen-activated protein kinase [MAPK] pathway), and the MAPK phosphatase MKP1 (via the extracellular signal-regulated kinase 1 and 2 [ERK1/2]–MAPK pathway) (3, 4).

Candidalysin is derived from a larger parental preproprotein (Ece1p) encoded by the *C. albicans ECE1* gene. The Ece1p preproprotein is thought to be processed in the endoplasmic reticulum (ER) and the Golgi complex and consists of 271 aa including a signal peptide for secretion (recognized by the signal peptidase) (5) and seven dibasic lysine-arginine (KR) motifs (see Fig. S1A in the supplemental material) that are recognized by the Golgi complex-associated endoproteinase Kex2p *in vitro* (6, 7). *C. albicans* Kex2p is a member of a family of eukaryotic proprotein protease enzymes that includes proprotein convertase 1 (PC1), PC2, and furin, which possess catalytic domains homologous to the degradative serine proteases of the subtilisin family (8). These subtilisin/kexin-like proteases have major physiological roles and are associated with various pathologies, including Alzheimer’s disease and tumorigenesis, and controlling infection in humans (9). Notably, the subtilisin/kexin-like proteases have been implicated in the activation of various bacterial toxins, including diphtheria toxin, *Pseudomonas aeruginosa* exotoxin A, botulinum neurotoxin, and pore-forming toxins such as aerolysin, which are produced as inactive, unprocessed forms that are activated by proteolytic processing (10). Therefore, we hypothesized that the processing of Ece1p by Kex2p to generate candidalysin may be required for *C. albicans* pathogenicity.

10.1128/mBio.02178-17.1 FIG S1  *C. albicans* Ece1p and candidalysin. (A) Amino acid sequence of *C. albicans* Ece1p. Lysine-arginine Kex2p recognition sequences (KR) are underlined and in bold. (B) Amino acid sequence of mature candidalysin with hydrophobic region (red) and hydrophilic region (green). (C) Model depicting sequential enzymatic processing of the candidalysin preproprotein (Ece1p). Ece1p (271 aa) is initially processed by the endoproteinase Kex2p, releasing immature candidalysin, SIIGIIMGILGNIPQVIQIIMSIVKAFKGNKR (Ece1p62–93). The carboxypeptidase Kex1p removes the C-terminal arginine residue from the immature toxin precursor to yield mature candidalysin, SIIGIIMGILGNIPQVIQIIMSIVKAFKGNK (Ece1p62–92). Download FIG S1, TIF file, 0.1 MB. Copyright © 2018 Richardson et al. 2018 Richardson et al. This content is distributed under the terms of the Creative Commons Attribution 4.0 International license.

Although recombinant Ece1p is a substrate of Kex2p-mediated proteolysis *in vitro* (6), it is not clear which KR motifs are important for targeted proteolysis of Ece1p, whether sequential Kex2p processing from the N or C terminus of Ece1p is required to release candidalysin, or whether Kex2p processing of Ece1p is critical for *in vivo* infections. To investigate the fundamental importance of Ece1p processing by Kex2p in *C. albicans* pathogenicity, it is not possible to use a *C. albicans kex2*Δ/Δ mutant as this mutant is unable to form hyphae and has severely attenuated fitness (11). We previously showed that a *kex2*Δ/Δ mutant is unable to damage and activate epithelial cells (3), but this cannot be attributed to defective Ece1p processing alone. To circumvent these problems, we used site-directed mutagenesis to mutate each of the Kex2p recognition sequences in Ece1p from lysine-arginine (KR) to lysine-alanine (KA), individually and in combination. Mutation of arginine to alanine replaces the charged polar side chain (Arg) with a smaller nonpolar side chain (Ala) and has been shown to reduce the efficiency of site-specific kexin-mediated proteolysis (12).

Using this approach, we demonstrate that Kex2p processing of Ece1p at Arg61 and Arg93 is vital for the generation of candidalysin, the induction of epithelial damage and immunity, and *C. albicans* pathogenicity. Furthermore, we show that sequential Kex2p processing from the N or C terminus of Ece1p is not required to release candidalysin. We also confirm that a second proteolytic processing event required for the production of mature candidalysin is dependent upon Kex1p, a carboxypeptidase that, like Kex2p, is associated with the Golgi complex in yeast (13). Collectively, these studies demonstrate that Arg61 and Arg93 of *C. albicans* Ece1p are indispensable residues required for a sequential, two-step processing event involving targeted proteolysis of Ece1p and candidalysin maturation and that this processing is required for *C. albicans* pathogenicity and immune activation.

## RESULTS

### Construction and characterization of alanine substitution mutants in *C. albicans* Ece1p.

To investigate the importance of the Kex2p recognition motifs within *C. albicans* Ece1p and their role in Ece1p processing, site-directed mutagenesis was used to mutate the arginine residue in each KR motif to an alanine (KA). The identity of each mutation was confirmed by DNA sequencing. The mutagenized constructs were introduced into a *C. albicans ece1*Δ/Δ null mutant (3) as the sole source of *ECE1* to create a panel of alanine substitution mutants (see Fig. S2 in the supplemental material) that allowed the importance of each KR site in Ece1p to be interrogated individually and in combination.

10.1128/mBio.02178-17.2 FIG S2  Schematic representation of wild-type and mutated Ece1p expressed from *C. albicans*. The region corresponding to candidalysin is shaded blue. The arginine residue in each of the seven individual KR Kex2p recognition motifs in Ece1p was mutated to alanine (red). Alanine substitutions that border candidalysin are underlined and in bold. Regions within Ece1p predicted to undergo inefficient cleavage as a consequence of alanine substitution are indicated by the dotted line. SP, signal peptide. Download FIG S2, TIF file, 0.1 MB. Copyright © 2018 Richardson et al. 2018 Richardson et al. This content is distributed under the terms of the Creative Commons Attribution 4.0 International license.

All mutants were viable and displayed no difference in growth rate in yeast extract-peptone-dextrose (YPD) liquid culture at 30°C. All mutants filamented normally under hypha-inducing conditions (RPMI 1640, 37°C, 3 h), and no significant differences in hyphal length were observed between the mutants and parental controls (Fig. S3A). The *ECE1* gene is strongly expressed during hyphal growth (3, 14). To confirm that expression of *ECE1* was not affected by the introduction of the alanine substitutions, we quantified the ability of each mutant to express *ECE1* under hypha-inducing conditions. No differences in *ECE1* expression were observed between any of the alanine substitution mutants or control strains (Fig. S3B).

10.1128/mBio.02178-17.3 FIG S3  Characterization of *C. albicans ECE1* mutants, Ece1p alanine substitution mutants and *KEX1* mutant strains. (A) Average hypha length of 50 fungal cells. Cells that did not germinate (remained in yeast morphology) were assigned a length of 0. Statistical significance was calculated using one-way ANOVA with a *post hoc* Dunnett comparison test + standard deviation (SD). Data represent *n* = 3 biological repeats. (B) Quantification of *C. albicans ECE1* gene expression normalized to the *ACT1* housekeeping gene and data presented as fold change relative to the yeast morphology of the reference strain SC5314. Statistical significance was calculated using one-way ANOVA with a *post hoc* Dunnett comparison test ± standard deviation (SD). Data represent *n* = 3 biological repeats. Download FIG S3, TIF file, 0.2 MB. Copyright © 2018 Richardson et al. 2018 Richardson et al. This content is distributed under the terms of the Creative Commons Attribution 4.0 International license.

### Ece1p Arg61 and Arg93 are essential for *C. albicans*-mediated epithelial damage and activation *in vitro.*

**FIG 1:**
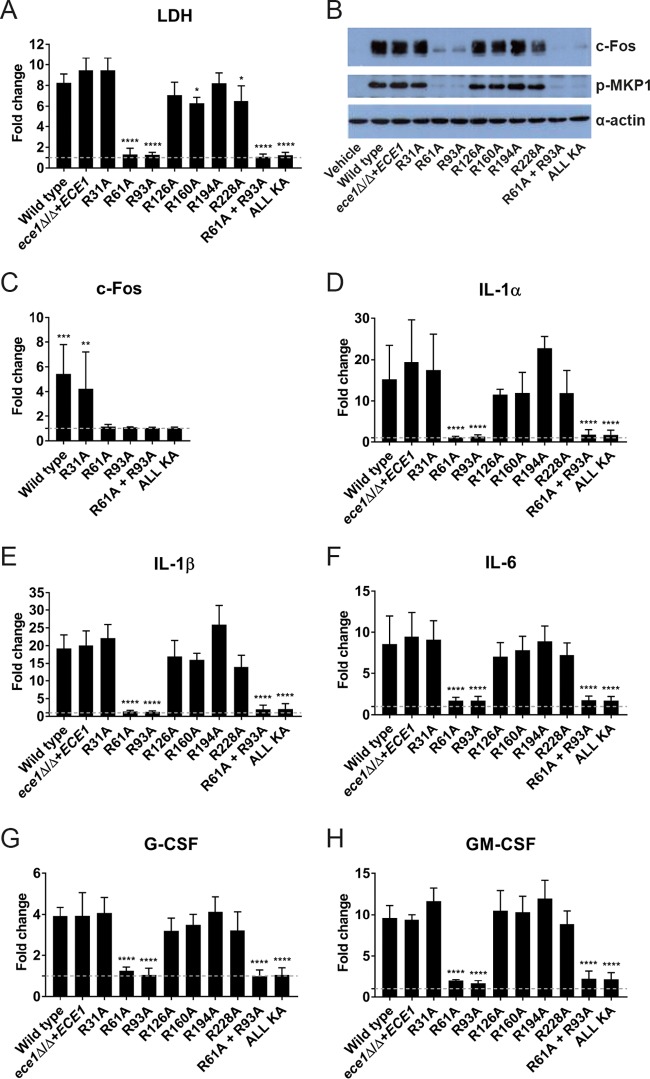
Alanine substitutions at positions 61 and 93 of Ece1p render *C. albicans* incapable of damaging or activating TR146 oral epithelial cells *in vitro*. (A) Epithelial cell damage induced by *C. albicans* Ece1p alanine substitution mutants. Epithelial cells were exposed to Ece1p alanine substitution mutants for 24 h, and levels of cell damage were assessed by LDH assay. Statistics are applied relative to the *ece1*Δ/Δ+*ECE1* parental control (*n* = 5 biological repeats). (B) Western blot analysis of epithelial cells infected with *C. albicans* alanine substitution mutants. Epithelial cell lysates (20 μg total protein) were probed with anti-c-Fos and anti-p-MKP1 antibodies. One representative blot presented (from *n* = 3 biological repeats). (C) Analysis of c-Fos DNA binding activity from epithelial cells infected with *C. albicans* alanine substitution mutants. Statistics are applied relative to the vehicle control (*n* = 3 biological repeats). (D to H) Quantification of cytokines (IL-1α, IL-1β, IL-6, G-CSF, and GM-CSF) secreted from epithelial cells in response to alanine substitution mutants of *C. albicans* Ece1p. Statistics are applied relative to *ece1*Δ/Δ+*ECE1* parental control (*n* = 3 biological repeats). (A and C to H) Data are presented as fold change relative to vehicle control (dashed line) + standard deviation (SD). Statistical significance was calculated using one-way ANOVA with a *post hoc* Dunnett comparison test. ****, *P* ≤ 0.0001; ***, *P* ≤ 0.001; **, *P* ≤ 0.01; *, *P* ≤ 0.05.

To determine whether processing of Ece1p by Kex2p is required for candidalysin-mediated damage to epithelial cells, we infected TR146 oral epithelial cells with the panel of alanine substitution mutants (Fig. S2) and quantified lactate dehydrogenase (LDH) activity in the culture supernatant as a marker of host cellular damage. Infection of epithelial cells with the isogenic wild-type strain (BWP17+CIp30, here referred to as “WT”) or the *ece1*Δ/Δ+*ECE1* parental control strain resulted in significant cellular damage compared to the vehicle (negative) control. Similarly, mutants expressing R31A, R126A, R160A, R194A, and R228A substitutions in Ece1p also caused significant damage. However, mutants expressing an alanine substitution at position 61 or 93 in Ece1p, and thus predicted to be unable to release candidalysin, were unable to damage epithelial cells (Fig. 1A). Strains expressing Ece1p containing both mutations (R61A + R93A) or all KR sites mutated to KA (ALL KA) were also unable to cause damage.

Candidalysin activates c-Fos and MKP1 signaling in epithelial cells, resulting in proinflammatory responses (3, 4, 15). To assess the role of Ece1p processing in signal pathway activation, epithelial cells were infected with the panel of Ece1p alanine substitution mutants *in vitro*, and c-Fos production/MKP1 phosphorylation was assessed by Western blotting. The c-Fos/p-MKP1 response was induced strongly by the WT, by the *ece1*Δ/Δ+*ECE1* parental control, and by strains expressing mutations that do not affect the release of candidalysin from Ece1p (R31A, R126A, R160A, R194A, and R228A). In contrast, epithelial cells infected with vehicle (negative control) or with R61A, R93A, R61A + R93A, or ALL KA mutant strains did not induce c-Fos production or MKP1 phosphorylation (Fig. 1B), and the lack of c-Fos activity was confirmed using a c-Fos DNA binding assay (Fig. 1C).

Candidalysin induces the secretion of several cytokines, including interleukin-1α (IL-1α), IL-1β, IL-6, granulocyte colony-stimulating factor (G-CSF) and granulocyte-macrophage colony-stimulating factor (GM-CSF) (3). Therefore, we quantified the secretion of these cytokines following infection with the panel of alanine substitution mutants (Fig. 1D to H). Epithelial cells responded to the WT, *ece1*Δ/Δ+*ECE1* parental control, and mutant strains that do not affect the release of candidalysin (R31A, R126A, R160A, R194A, and R228A), by secreting significant levels of IL-1α, IL-1β, IL-6, G-CSF, and GM-CSF. In contrast, epithelial cytokine secretion was abolished in response to infection with the mutant strains predicted to affect the release of candidalysin (R61A, R93A, R61A + R93A, and ALL KA). Taken together, these data indicate that Arg61 and Arg93 within *C. albicans* Ece1p are essential for the release of candidalysin and the induction of epithelial damage and immune activation *in vitro*.

### R61A and R93A mutations in *C. albicans* Ece1p result in attenuated secretion of candidalysin.

To confirm whether mutation of Kex2p recognition sites within Ece1p impaired candidalysin secretion, hyphal growth was induced in mutant and control strains, and hypha-secreted peptides were analyzed by liquid chromatography-tandem mass spectrometry (LC-MS/MS). The number of peptide spectrum matches (PSMs) corresponding to candidalysin was determined (Table 1; amino acid sequences of detected peptides and nomenclature conventions are presented in Table S1 in the supplemental material).

10.1128/mBio.02178-17.5 TABLE S1  Nomenclature and amino acid sequence of Ece1p peptides. Download TABLE S1, DOCX file, 0.01 MB. Copyright © 2018 Richardson et al. 2018 Richardson et al. This content is distributed under the terms of the Creative Commons Attribution 4.0 International license.

**TABLE 1:** LC-MS/MS analysis of Ece1p secreted peptides^e^

Strain name^a^	Conditions and replicate^b^	Total no. of PSMs^c^	No. of PSMs for candidalysin^c^	Most abundant sequence^d^ (no. of PSMs)
SC5314	(1)	815	197	P7N_DT-19 (268)
	(2)	416	189	Candidalysin
BWP17+CIp30	(1)	2,011	715	Candidalysin
	(2)	1,576	730	Candidalysin
	(3)	112	79	Candidalysin
	3 h culture	595	422	Candidalysin
	TR146 epithelial cell infection, 3 h (1)	154	128	Candidalysin
	TR146 epithelial cell infection, 3 h (2)	81	79	Candidalysin
	TR146 epithelial cell infection, 18 h	209	97	Candidalysin
	Pepstatin A	794	335	Candidalysin
	Pepstatin A vehicle control	1,661	966	Candidalysin
*ece1*Δ/Δ	(1)	0	0	NA
	(2)	0	0	NA
*ece1Δ/Δ+ECE1*		1,090	510	Candidalysin
*ece1Δ/Δ+ECE1*_Δ184−279_		188	0	P7C_SV-12 (46)
*kex1*Δ/Δ	(1)	2,681	80	P8N_DK-13 (604)
	(2)	1,369	49	P8N_DK-13 (338)
	(3)	559	19	P7N_DR-20 (140)
*kex1Δ/Δ+KEX1*		468	110	P7N_DT-19 (228)
R31A	(1)	489	154	Candidalysin
	(2)	483	273	Candidalysin
R61A	(1)	1,441	92	P2-P3K_DK-61 (370)
	(2)	179	3	P2-P3K_DK-61 (78)
R93A	(1)	338	15	P7C_SV-12 (108)
	(2)	90	0	P7N_DT-19 (26)
R126A	(1)	371	133	Candidalysin
	(2)	323	213	Candidalysin
R160A		443	146	P7N_DT-19 (189)
R194A		1,169	752	Candidalysin
R228A		418	276	Candidalysin
R61A + R93A	(1)	464	0	P7N_DT-19 (200)
	(2)	127	4	P7N_SV-12 (35)
ALL KA		186	0	P5N_DA-19 and P8C_DA-13 (20)

^a^ Genotype details are presented in Table S3.

^b^ Numbers in parentheses indicate independent experimental replicates.

^c^ PSM values are semiquantitative.

^d^ Amino acid sequences of detected peptides and nomenclature conventions are presented in Table S1. PSM, peptide spectrum match; NA, not applicable. Full details of LC-MS/MS data sets and sequence alignments are provided in Data Set S1.

^e^ Samples were prepared as previously described (3) unless otherwise specified.

The *C. albicans* reference strain SC5314 (16) and the isogenic WT strain BWP17+CIp30 secreted candidalysin in the absence of epithelial cells under hypha-inducing conditions, and BWP17+CIp30 secreted candidalysin when cultured in the presence of epithelial cells for 3 h and 18 h. No candidalysin PSMs were detected from an *ece1*Δ/Δ null mutant or a mutant lacking the candidalysin-encoding region of *ECE1* (*ece1*Δ/Δ+*ECE1*_Δ184–279_). Substantial candidalysin PSMs were obtained from the *ece1*Δ/Δ+*ECE1* parental control, and candidalysin was the most abundant peptide detected in the strains harboring alanine substitutions that did not flank candidalysin (R31A, R126A, R194A, and R228A; the exception was the R160A strain, where candidalysin was the second most abundant peptide). In contrast, secretion of candidalysin from strains that harbored alanine substitutions which flanked candidalysin (R61A, R93A, R61A + R93A, and ALL KA) was absent or severely attenuated (Table 1). These data demonstrate that Kex2p recognition of arginine residues at positions 61 and 93 within Ece1p is required for efficient release of candidalysin.

LC-MS/MS analysis of peptides secreted from R61A and R93A mutants demonstrated that while candidalysin (SIIGIIMGILGNIPQVIQIIMSIVKAFKGNK) displayed minimal PSM values relative to total PSMs (Table 1), larger “fusion” peptides containing the mutated Kex2p recognition site (KA) were detected in the exhausted culture medium (Data Set S1), suggesting that Kex2p was unable to process Ece1p at these mutated locations. Fusion peptides were secreted from all alanine substitution mutants except the R126A and R228A mutants. Collectively, these data indicate that the KR motifs of Ece1p are required for enzymatic processing of the full-length protein into peptide fragments and that Arg61 and Arg93 are critical for the secretion of candidalysin.

10.1128/mBio.02178-17.9 DATA SET S1  LC-MS/MS analysis of hypha-secreted Ece1p peptides. Raw LC-MS/MS data are provided as well as a peptide alignment to the complete Ece1 sequence. Predicted amino acid sequence of peptides produced following Kex2p digestion of Ece1p *in vitro* (red). Amino acid sequence of secreted peptides detected by LC-MS/MS (black) and peptide spectrum match (PSM) values (brackets). Only peptides that reach an arbitrary threshold of ≥10 PSMs are shown. Peptides that have lower PSM values can be found above. Download DATA SET S1, XLSX file, 0.4 MB. Copyright © 2018 Richardson et al. 2018 Richardson et al. This content is distributed under the terms of the Creative Commons Attribution 4.0 International license.

### *C. albicans* Kex1p is required for candidalysin maturation, epithelial damage, and immune activation.

The candidalysin toxin was initially predicted to terminate in a dibasic lysine-arginine (KR) motif based upon Kex2p substrate specificity, and a peptide corresponding to this sequence (SIIGIIMGILGNIPQVIQIIMSIVKAFKGN**KR**) was capable of damaging epithelial cells and activating the c-Fos/MKP1 signaling circuits *in vitro* (3). However, subsequent analysis of the hypha-secreted peptides revealed that secreted candidalysin lacks the C-terminal arginine residue (3). This observation implied the potential involvement of a carboxypeptidase enzyme and a second processing step. In addition to the Kex2p endoproteinase, the carboxypeptidase Kex1p is also located in the Golgi complex of yeast (13), and an ortholog of Kex1p exists in *C. albicans*. Given these observations, Kex1p was therefore a compelling candidate for the removal of the C-terminal arginine residue and production of mature candidalysin and was confirmed to do so in a previous study (3).

Further analysis of the hypha-secreted peptides of a *kex1*Δ/Δ null mutant (Table 1) revealed an approximately 7-fold reduction in candidalysin PSM values relative to total PSM values compared with a *kex1*Δ/Δ+*KEX1* reintegrant control (3.2% versus 23.5%, respectively). Importantly, analysis of WT and the *kex1*Δ/Δ+*KEX1* strain revealed that mature candidalysin (SIIGIIMGILGNIPQVIQIIMSIVKAFKGN**K**) was the predominant peptide secreted from both strains. However, upon disruption of the *KEX1* gene (*kex1*Δ/Δ), immature candidalysin (SIIGIIMGILGNIPQVIQIIMSIVKAFKGN**KR**) was the dominant peptide produced (Table S2). Collectively, these data confirm that *C. albicans* Kex1p carboxypeptidase activity is required for the production of mature candidalysin.

10.1128/mBio.02178-17.6 TABLE S2  LC-MS/MS analysis of *kex1*Δ/Δ hypha-secreted Ece1p peptides; the role of Kex1p in candidalysin maturation. Download TABLE S2, DOCX file, 0.01 MB. Copyright © 2018 Richardson et al. 2018 Richardson et al. This content is distributed under the terms of the Creative Commons Attribution 4.0 International license.

To determine whether *C. albicans* Kex1p activity was required for cellular damage, signal pathway activation, and the induction of proinflammatory cytokines, we infected TR146 epithelial monolayers with a *kex1*Δ/Δ null mutant. In contrast to the WT and *kex1*Δ/Δ+*KEX1* reintegrant control strains, the *kex1*Δ/Δ null mutant was unable to cause damage or induce c-Fos production/DNA binding, MKP1 phosphorylation, or cytokine secretion from epithelial cells (Fig. S4). These data demonstrate that Kex1p processing is also required for epithelial damage and host recognition of candidalysin.

10.1128/mBio.02178-17.4 FIG S4  *C. albicans* Kex1p is required for damage and activation of TR146 oral epithelial cells *in vitro*. (A) Epithelial cell damage induced by *C. albicans* WT (parental control), *kex1*Δ/Δ strain, and *kex1*Δ/Δ+*KEX1* reintegrant. Epithelial cells were exposed to *C. albicans* strains for 24 h, and levels of cell damage were assessed by LDH assay. Data are presented as fold change relative to vehicle control (dashed line; *n* = 3 biological repeats) + standard deviation (SD). (B) Western blot analysis of epithelial cells infected with *C. albicans* WT (parental control), *kex1*Δ/Δ strain, and *kex1*Δ/Δ+*KEX1* reintegrant. Epithelial cell lysates (20 μg total protein) were probed with anti c-Fos and anti-p-MKP1 antibodies. One representative blot presented (from *n* = 3 biological repeats). (C) Analysis of c-Fos DNA binding activity in epithelial cells infected with *C. albicans* WT (parental control), *kex1*Δ/Δ strain, and *kex1*Δ/Δ+*KEX1* reintegrant. Data are presented as fold change relative to vehicle control (dashed line; *n* = 4 biological repeats) + SD. (D to H) Quantification of cytokines (IL-1α, IL-1β, IL-6, G-CSF, and GM-CSF) secreted from epithelial cells in response to *C. albicans* WT (parental control), *kex1*Δ/Δ strain, and *kex1*Δ/Δ+*KEX1* reintegrant. Data are presented as fold change relative to vehicle control (dashed line; *n* = 3 biological repeats) + SD. Statistics are applied relative to *kex1*Δ/Δ+*KEX1* parental control. (A and C to H) Statistical significance was calculated using one-way ANOVA with a *post hoc* Dunnett comparison test. ****, *P* ≤ 0.0001; ***, *P* ≤ 0.001; **, *P* ≤ 0.01; *, *P* ≤ 0.05. Download FIG S4, TIF file, 1.1 MB. Copyright © 2018 Richardson et al. 2018 Richardson et al. This content is distributed under the terms of the Creative Commons Attribution 4.0 International license.

### Saps are not required for Ece1p processing or candidalysin production.

Like Kex2p, Kex1p is likely to have multiple targets, including other proteases. The observations made with the *kex1*Δ/Δ null mutant could therefore be an indirect effect of the absence of Kex1p, which could be required for full activity of other proteases possibly involved in the maturation of candidalysin. The secreted aspartyl proteinases (Saps) are a family of enzymes that exhibit broad substrate specificity (17). Pepstatin A is a potent inhibitor of most Sap activity (although Sap7p is not inhibited by pepstatin A, and Sap9p and Sap10p exhibit reduced sensitivity to such inhibition under certain physiological conditions [18, 19]), and epithelial cells treated with pepstatin A are partially protected from fungal damage *in vitro* (20). In light of these observations, we investigated whether Saps may play a role in Ece1p processing and candidalysin production. Therefore, we cultured WT *C. albicans* under hypha-inducing conditions in the presence of pepstatin A and analyzed the secreted peptides using LC-MS/MS (Table 1). Candidalysin was secreted from WT hyphae in the presence of pepstatin A, and no reduction in candidalysin PSMs was observed following Sap inhibition. These observations suggest that Saps and/or other extracellular aspartyl proteinases (e.g., Bar1p [21]) targeted by pepstatin A are not required for Ece1p processing or candidalysin production, at least when inhibited extracellularly.

### Arg93 is required for efficient processing of *C. albicans* Ece1p.

Candidalysin possesses a hydrophobic N-terminal region (Ece1p_62–85_; SIIGIIMGILGNIPQVIQIIMSIV) and a hydrophilic C terminus (Ece1p_86−92_; KAFKGNK) (Fig. S1B). A previous *in vitro* analysis of mutated candidalysin peptides indicated that the positively charged C terminus of candidalysin is required for epithelial damage (3). Having demonstrated that Arg61 and Arg93 are required for efficient enzymatic processing of Ece1p, we questioned whether replacement of arginine with alanine at position 93 could nevertheless result in successful (albeit inefficient) proteolytic cleavage of mutated Ece1p, generating a secreted candidalysin toxin with a modified C terminus (amino acid sequence SIIGIIMGILGNIPQVIQIIMSIVKAFKGNK**A**; Ece1p62–93_KA_). LC-MS/MS analysis of hypha-secreted peptides from the R93A mutant revealed the presence of this modified candidalysin toxin terminating in lysine-alanine (Data Set S1). However, the PSM value for this modified toxin was very low (PSM = 14; complete LC-MS/MS data set is provided in Data Set S1), and infection of epithelial cells with the R93A mutant did not induce cellular damage, MKP1 phosphorylation, c-Fos DNA binding, or cytokine secretion (Fig. 1).

**FIG 2:**
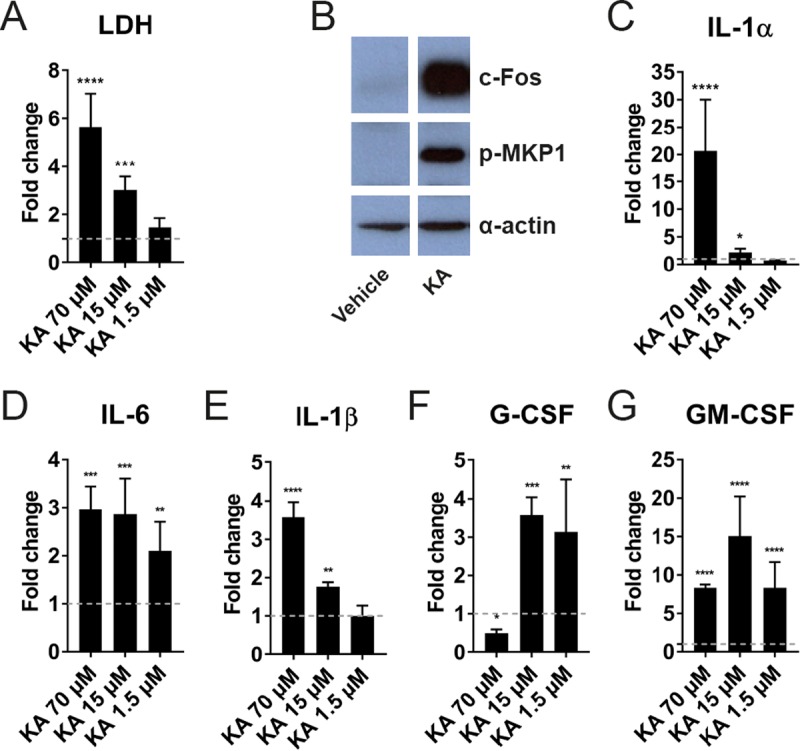
Ece1p62–93_KA_ damages epithelial cells and activates c-Fos/p-MKP1 signaling and cytokine secretion *in vitro*. (A) Epithelial cell damage induced by Ece1p62–93_KA_. Epithelial cells were exposed to Ece1p62–93_KA_ peptide (70, 15, and 1.5 μM) for 24 h, and levels of cell damage were assessed by LDH assay. (B) Western blot analysis of epithelial cells treated with Ece1p62–93_KA_ peptide (15 μM) for 2 h. Epithelial cell lysates (20 μg total protein) were probed with anti-c-Fos and anti-p-MKP1 antibodies. One representative blot is presented (from *n* = 3 biological repeats). (C to G) Quantification of cytokines (IL-1α, IL-1β, IL-6, G-CSF, and GM-CSF) secreted from epithelial cells in response to Ece1p62–93_KA_ peptide at 70, 15, and 1.5 μM. (A and C to G) Data and statistical analysis are presented relative to vehicle control (dashed line; *n* = 3 biological repeats) + standard deviation (SD). Statistical significance was calculated using one-way ANOVA with a *post hoc* Dunnett comparison test. ****, *P* ≤ 0.0001; ***, *P* ≤ 0.001; **, *P* ≤ 0.01; *, *P* ≤ 0.05.

To determine whether direct application of Ece1p62–93_KA_ was capable of causing epithelial damage and immune activation, we treated TR146 cells with different concentrations (70, 15, and 1.5 μM; ranging from lytic to sublytic concentrations of mature candidalysin [3]) of Ece1p62–93_KA_ peptide and quantified LDH activity, c-Fos/p-MKP1 responses, and cytokine secretion (Fig. 2). At lytic concentrations, Ece1p62–93_KA_ peptide caused dose-dependent epithelial damage (Fig. 2A) and induced c-Fos and p-MKP1 responses (Fig. 2B) and secretion of cytokines (Fig. 2C to G). These data affirm the ability of Ece1p62–93_KA_ to cause epithelial damage and immune activation *in vitro* and suggest that the lack of epithelial damage and signaling observed in response to the R93A mutant arose from inefficient processing of Ece1p resulting in a severe reduction in Ece1p62–93_KA_ secretion.

### Ece1p processing is required for the induction of mucosal immune responses *in vivo.*

**FIG 3:**
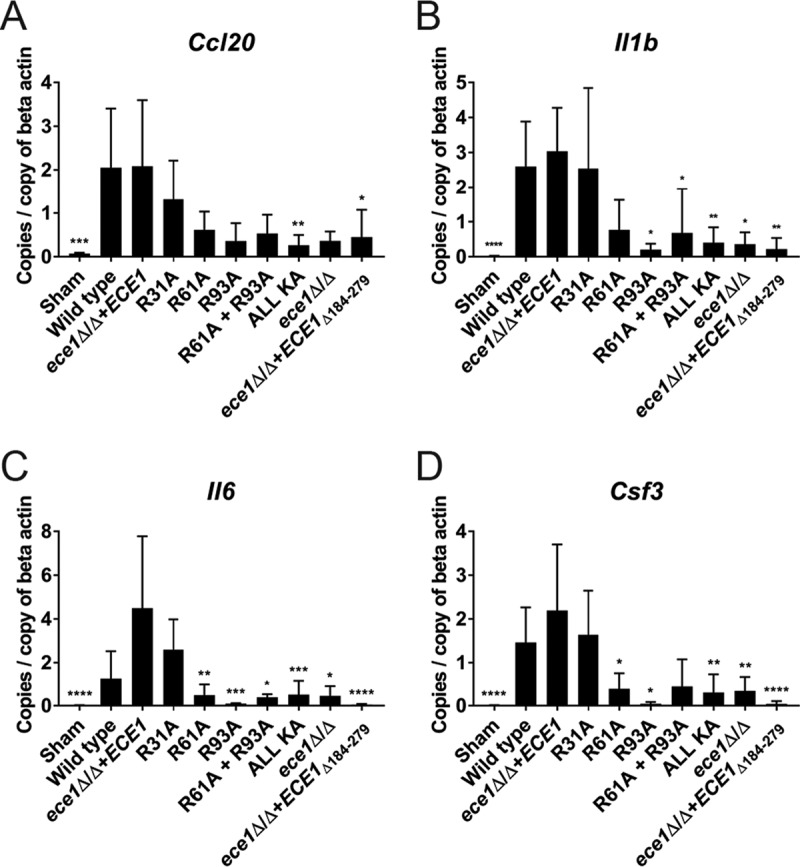
Enzymatic processing of *C. albicans* Ece1p is required for efficient host recognition of infecting fungus *in vivo*. Nonimmunosuppressed mice were infected sublingually with selected *C. albicans* Ece1p alanine substitution mutants and control strains for 24 h, and reverse transcriptase qPCR was performed on tongue tissue RNA to quantify expression of *Ccl20* (A), *Il1b* (B), *Il6* (C), and *Csf3* transcripts (D). Data are presented as the mean of two biological repeats + standard deviation (SD). Statistical analysis is presented relative to the *ece1*Δ/Δ+*ECE1* parental control (total numbers of animals: sham, *n* = 4; wild type, *n* = 4; *ece1*Δ/Δ+*ECE1* strain, *n* = 6; R31A, *n* = 6; R61A, *n* = 5; R93A, *n* = 3; R61A + R93A, *n* = 7; ALL KA, *n* = 7; *ece1*Δ/Δ strain, *n* = 5; *ece1*Δ/Δ+*ECE1*_Δ184–279_ strain, *n* = 5). Statistical significance was calculated using one-way ANOVA with a *post hoc* Dunnett comparison test. ****, *P* ≤ 0.0001; ***, *P* ≤ 0.001; **, *P* ≤ 0.01; *, *P* ≤ 0.05.

Mucosal responses to infiltrating pathogenic *C. albicans* hyphae culminate in the secretion of immunomodulatory cytokines and chemokines that collectively drive innate immune responses leading to fungal clearance (reviewed in reference 22). To determine whether enzymatic processing of *C. albicans* Ece1p is a driver of early-phase host immune responses *in vivo*, we used a nonimmunosuppressed murine model of oropharyngeal candidiasis (OPC). WT mice were infected with positive-control strains (WT and *ece1*Δ/Δ+*ECE1*), negative-control strains (*ece1*Δ/Δ and *ece1*Δ/Δ+*ECE1*_Δ184–279_), a candidalysin-secreting alanine substitution mutant (R31A), and alanine substitution mutants that showed a marked reduction in candidalysin secretion (R61A, R93A, R61A + R93A, and ALL KA) for 24 h. Gene expression of *Ccl20*, *Il1b*, *Il6*, and *Csf3* from infected tongue tissue was assessed by quantitative PCR (qPCR) (Fig. 3).

Mice infected with the WT or *ece1*Δ/Δ+*ECE1* strain or a candidalysin-secreting alanine substitution mutant (R31A) responded by expressing increased levels of *Ccl20*, *Il1b*, *Il6*, and *Csf3* compared to the vehicle (negative) control (Fig. 3A to D). In contrast, mice infected with the R61A, R93A, R61A + R93A, and ALL KA mutants were significantly attenuated in their ability to induce the expression of at least one proinflammatory gene compared with the *ece1*Δ/Δ+*ECE1* parental control. Indeed, the impaired response was similar to that of negative-control strains that did not express *ECE1* (*ece1*Δ/Δ) or that lacked the candidalysin-encoding region of the *ECE1* gene (*ece1*Δ/Δ+*ECE1*_Δ184–279_). These data confirm the importance of Ece1p processing and the secretion of candidalysin for the early-phase activation of mucosal immune responses to *C. albicans* hyphae *in vivo*.

### Arg61 and Arg93 of *C. albicans* Ece1p are required for mucosal infection *in vivo.*

**FIG 4:**
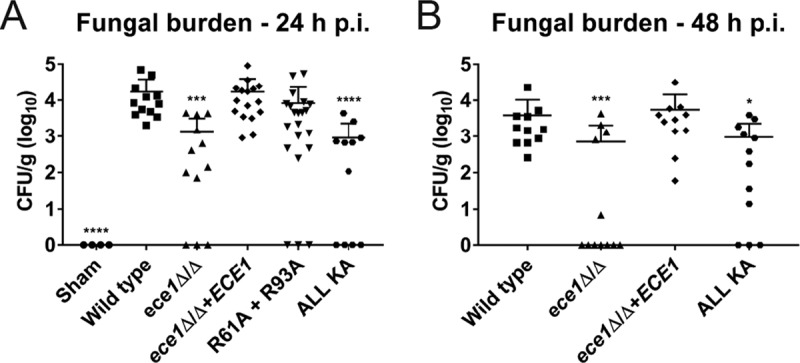
Enzymatic processing of *C. albicans* Ece1p is required for fungal pathogenesis *in vivo*. An immunosuppressed murine model of oropharyngeal candidiasis (OPC) was infected with selected *C. albicans* Ece1p alanine substitution mutants and control strains, and the number of CFU per gram of tissue was enumerated after 24 h (A) and 48 h (B). Data represent two biological repeats. Total numbers of animals in panel A: sham, *n* = 4; wild type, *n* = 12; *ece1*Δ/Δ strain, *n* = 12; *ece1*Δ/Δ+*ECE1* strain, *n* = 16; R61A + R93A, *n* = 21; ALL KA, *n* = 11. Total numbers of animals in panel B: wild type, *n* = 11; *ece1*Δ/Δ strain, *n* = 12; *ece1*Δ/Δ+*ECE1* strain, *n* = 11; ALL KA, *n* = 12. Statistical analysis is presented relative to the *ece1*Δ/Δ+*ECE1* parental control. Statistical significance was calculated using one-way ANOVA with a *post hoc* Dunnett comparison test. Geometric mean is indicated. ****, *P* ≤ 0.0001; ***, *P* ≤ 0.001; *, *P* ≤ 0.05.

A *C. albicans ece1*Δ/Δ mutant and a mutant lacking the candidalysin-encoding region of *ECE1* (*ece1*Δ/Δ+*ECE1*_Δ184–279_) are severely diminished in their ability to cause murine OPC and disease in a zebrafish swim bladder model of mucosal infection (3). To determine whether processing of Ece1p at Arg61 and Arg93 was required for successful fungal infection *in vivo*, we challenged immunosuppressed WT mice with positive-control strains (WT and *ece1*Δ/Δ+*ECE1*), negative-control strains (*ece1*Δ/Δ), and selected alanine substitution mutants (R61A + R93A and ALL KA) and quantified fungal burdens in tongue tissue after 24 or 48 h. Mice infected with the WT or *ece1*Δ/Δ+*ECE1* parental control strain exhibited high levels of fungal burdens compared to sham-infected (negative) controls after 24 h (Fig. 4A). Mice infected with the ALL KA mutant produced a statistically significant reduction in fungal burdens consistent with the *ece1*Δ/Δ mutant. The R61A + R93A mutant also exhibited a (nonsignificant) reduction in fungal burdens compared to the *ece1*Δ/Δ+*ECE1* parental control. Repeat experiments at 48 h showed that the ALL KA mutant (the R61A + R93A mutant was not tested) maintained a significant reduction in fungal burdens compared with the *ece1*Δ/Δ+*ECE1* strain (Fig. 4B). Taken together, these data demonstrate the importance of Ece1p processing in *C. albicans* pathogenicity in oral infections.

## DISCUSSION

Subtilisin/kexin-like proteases have fundamental physiological roles in nature and have been associated with various pathologies, including Alzheimer’s disease, tumorigenesis, and the activation of multiple bacterial toxins (9, 10). The human fungal pathogen *C. albicans* also possesses a Kex2p-like endoproteinase, and given that Ece1p (the preproprotein harboring the peptide toxin candidalysin) is a known substrate for Kex2p *in vitro* (6), this study investigated the importance of Ece1p processing for the production of candidalysin and *C. albicans* pathogenicity.

The endoproteinase Kex2p cleaves protein substrates and model peptides after dibasic arginine-arginine (RR) and lysine-arginine (KR) motifs (8). Analysis of the *C. albicans* genome has identified 147 potential Kex2p substrates, including Saps, the hypha-wall protein Hwp1p (7), and Ece1p (6). However, a *kex2*Δ/Δ null mutant has severely attenuated fitness (11) and is unable to damage and activate epithelial cells due to its inability to form hyphae (3). Since hypha formation is strongly associated with *ECE1* gene expression (3, 14), the lack of epithelial cell activation by the *kex2*Δ/Δ null mutant cannot be attributed to defective Ece1p processing alone. Therefore, to address the importance of Kex2p for Ece1p processing, we created a panel of alanine substitution mutants in Ece1p in which each Kex2p recognition site (KR) was mutated, individually or in combination. With this approach, we were able to circumvent any off-target effects imposed by *KEX2* disruption and address three outstanding questions regarding Ece1p processing and candidalysin production: (i) which KR motifs are important for candidalysin production and maturation, (ii) whether sequential Kex2p processing from the N or C terminus of Ece1p is required for candidalysin release, and (iii) whether Ece1p processing by Kex2p is critical for *C. albicans* pathogenicity and mucosal infection.

*C. albicans* mutants (R61A, R93A, R61A + R93A, and ALL KA) harboring replacements of Arg61 and Arg93 with alanine within Ece1p, which directly flank candidalysin (see Fig. S2 in the supplemental material), were unable to induce epithelial damage, c-Fos production, DNA binding, MKP1 phosphorylation, and cytokine secretion *in vitro* (Fig. 1), which are key readouts of candidalysin activity (3). The same inability to induce these phenotypes was also observed in *C. albicans* mutants lacking the *ECE1* gene (*ece1*Δ/Δ) (Fig. 1) or the candidalysin-encoding region of *ECE1* (*ece1*Δ/Δ+*ECE1*_Δ184–279_) (3). Notably, mutation of arginine residues that did not directly flank candidalysin (R31A, R126A, R160A, R194A, and R228A) failed to abrogate these responses. LC-MS/MS analysis of hypha-secreted peptides revealed that mutations at Arg61 and Arg93 markedly reduced candidalysin secretion, whereas mutations at all other arginine residues did not prevent secretion (Table 1). These data demonstrate that only the Arg residues that directly flank the candidalysin region (Arg61 and Arg93) are required for the release of candidalysin from Ece1p and that these processing events are essential for the ability of *C. albicans* hyphae to cause epithelial damage and immune activation. Importantly, the data also demonstrate that sequential Kex2p processing from the N or C terminus of Ece1p is not required for the release of candidalysin from the Ece1p preproprotein.

Analysis of hypha-secreted peptides demonstrated that, in all cases, mutants with an altered KA motif still secreted the modified fusion peptide into the extracellular milieu (Table 1), strongly suggesting that secretion pathways are intact in all of these strains. Likewise, the fact that candidalysin was secreted from R31A, R126A, R160A, R194A, and R228A mutants (Table 1) indicates that candidalysin release from Ece1p is dependent upon processing at Arg61 and Arg93 alone and is not influenced by mutations of Kex2p recognition sites that do not directly border the toxin.

Mature candidalysin (terminating in K) is the predominant toxin secreted from WT *C. albicans*. Given that the endoproteinase Kex2p cleaves protein substrates after lysine-arginine (KR) motifs (8), it became apparent that a second cleavage event was occurring that resulted in the removal of the C-terminal arginine residue from immature candidalysin. The removal of C-terminal arginine residues from proteins and peptides is the function of the carboxypeptidase Kex1p (23–25). To confirm that removal of the C-terminal arginine residue from immature candidalysin was due to the function of Kex1p (3), we analyzed the hypha-secreted peptides of a *kex1*Δ/Δ null mutant compared to its matched revertant strain. The dominant peptide secreted from the *kex1*Δ/Δ null mutant was immature candidalysin (terminating in KR), whereas the *kex1*Δ/Δ+*KEX1* revertant showed WT-like secretion patterns (Table S2). This demonstrates that Kex1p activity is an important requirement for candidalysin maturation. However, the biological reason for removal of the C-terminal arginine residue by Kex1p is unclear, as both immature (Ece1-III_62–93KR_) and mature (Ece1-III_62–92K_) candidalysin are able to damage and activate epithelial cells, with Ece1-III_62–93KR_ being even more cytolytic than mature candidalysin at lower concentrations (3). We thus postulate that the removal of the C-terminal arginine must confer an evolutionary advantage to *C. albicans*, either as a commensal or as a pathogen. Furthermore, it is likely that Kex1p (like Kex2p) targets multiple proteins and peptides in addition to immature candidalysin, which are also required for fungal fitness and virulence. These observations suggest that the immature candidalysin secreted from *C. albicans kex1*Δ/Δ null mutant hyphae is not present in sufficient concentrations to cause damage to host epithelial cells if other fungal attributes are dysfunctional. This could account for the attenuated damage potential of the *kex1*Δ/Δ null mutant.

Exhausted culture medium from epithelial cells infected with WT *C. albicans* failed to induce detectable damage on freshly cultured epithelial cells (not shown). The most likely explanation for this observation is that the concentration of candidalysin secreted into the extracellular environment is insufficient to cause plasma membrane destabilization. Indeed, we propose that in addition to correct processing of *C. albicans* Ece1p, an epithelial invasion pocket produced by an invading hypha is also required in order for secreted candidalysin to reach the concentrations necessary to cause epithelial damage (3).

We also questioned whether extracellular aspartyl proteases, including Saps (17) and Bar1p (21), could be involved in Ece1p processing and/or candidalysin maturation. Culture of WT *C. albicans* under hypha-inducing conditions in the presence of the aspartyl protease inhibitor pepstatin A followed by LC-MS/MS analysis of the hypha-secreted peptides revealed that candidalysin secretion was unaffected (Table 1). Furthermore, *C. albicans* mutant strains unable to express *SAP2*, *SAP7*, and *SAP9/10* were observed to cause epithelial damage and activation (3), suggesting that processing of Ece1p and secretion of candidalysin were unaffected by these enzymes.

To determine whether defective Ece1p processing impacted *C. albicans* pathogenicity *in vivo*, we utilized a murine model of OPC. Since mice are immunologically naive to *C. albicans*, we first investigated the ability of selected substitution mutants to induce proinflammatory gene expression in the tongue tissue of immunocompetent mice. Only those *C. albicans* mutants harboring substitutions at Arg61 and Arg93 (R61A, R93A, R61A + R93A, and ALL KA), which flank candidalysin, were severely diminished in their ability to induce early-phase (24 h) gene expression, similar to the *ece1*Δ/Δ mutant (Fig. 3). This demonstrates that Ece1p processing at Arg61 and Arg93 and subsequent candidalysin secretion are crucial for the induction of immune responses against *C. albicans in vivo*. Given that *C. albicans* is not a natural colonizer of mice, we next investigated the ability of the R61A + R93A and ALL KA substitution mutants to colonize tongue tissue in an immunosuppressed OPC model. Both mutants showed a reduced capacity to infect tongue tissue at 24 h, with significant reductions in fungal burdens observed with the ALL KA substitution mutant (Fig. 4A). Additional experiments with the ALL KA substitution mutant indicated that fungal burdens remained low after 48 h (Fig. 4B). We noted that while the R61A + R93A and ALL KA mutants induced almost identical epithelial phenotypes *in vitro* (Fig. 1), they induced subtly different phenotypes in the context of OPC *in vivo* after 24 h (Fig. 4A), with the ALL KA mutant exhibiting a greater reduction in fungal burdens than the R61A + R93A mutant. This raises the possibility that candidalysin may have some residual activity when fused to an adjacent peptide but not when contained within full-length Ece1p. Alternatively, Ece1p processing at sites other than Arg61 and Arg93, and hence other Ece1p-derived peptides, may have a role in fungal pathogenesis *in vivo*. Indeed, the role (if any) of the other noncandidalysin peptides derived from Kex2p-Kex1p processing of Ece1p remains to be determined. Investigations are under way to address this question.

In summary, this study demonstrates that the sequential two-step posttranslational processing of *C. albicans* Ece1p by kexin-like proteinases is a critical event required for candidalysin production, immune activation, and *C. albicans* pathogenicity. Site-specific proteolytic degradation of Ece1p by the endoproteinase Kex2p releases immature candidalysin, SIIGIIMGILGNIPQVIQIIMSIVKAFKGNKR (Ece1p_62−93_), terminating with a C-terminal arginine (Arg93). Following release from Ece1p, the immature toxin is further processed by the carboxypeptidase Kex1p, which removes a C-terminal arginine to yield mature candidalysin, SIIGIIMGILGNIPQVIQIIMSIVKAFKGNK (Ece1p_62−92_) (Fig. S1C). The release of candidalysin from Ece1p can now be added to a growing list of protein zymogens and peptide precursors that are targeted for proteolysis to produce smaller biologically active molecules, including fungal killer toxins (23), hydrophobic plant repellent peptides (26), mating pheromones (27), and mammalian prohormones (28). Given the fundamental role of candidalysin in *C. albicans* virulence and the presence of kexin-like proteinases in other human-pathogenic fungi, kexin-mediated processing events and/or candidalysin itself may provide novel targets for the development of new therapeutic drugs to treat fungal infections.

## MATERIALS AND METHODS

### Fungal strains.

All fungal strains used in this study are presented in Table S3 in the supplemental material (3, 16, 29, 30).

10.1128/mBio.02178-17.7 TABLE S3  *Candida albicans* strains used in this study. Download TABLE S3, DOCX file, 0.01 MB. Copyright © 2018 Richardson et al. 2018 Richardson et al. This content is distributed under the terms of the Creative Commons Attribution 4.0 International license.

### Oligonucleotides.

Oligonucleotide primers were purchased from Integrated DNA Technologies (Belgium). The sequence of primers used in this study is provided in Table S4.

10.1128/mBio.02178-17.8 TABLE S4  Oligonucleotide primers used in this study. Download TABLE S4, DOCX file, 0.02 MB. Copyright © 2018 Richardson et al. 2018 Richardson et al. This content is distributed under the terms of the Creative Commons Attribution 4.0 International license.

### Peptides.

Peptides were purchased from Peptide Protein Research Ltd. (UK).

### Antibodies.

p-DUSP1/MKP1 (S359) and c-Fos rabbit monoclonal antibodies were purchased from Cell Signaling Technologies (catalog numbers 2857 and 2250, respectively). Actin (clone C4) mouse monoclonal antibody was purchased from Millipore (catalog number MAB1501). Peroxidase-conjugated AffiniPure goat anti-mouse and anti-rabbit IgG secondary antibodies were purchased from Jackson Immune Research (catalog numbers 115-035-062 and 111-035-003, respectively).

### Mammalian cell culture.

Experiments were performed using the TR146 human oral epithelial cell line (31) (purchased from the European Collection of Authenticated Cell Cultures). Cells were cultured in Dulbecco modified Eagle medium (DMEM)–F-12 nutrient mixture (1:1) plus l-glutamine (Life Technologies) supplemented with 15% (vol/vol) heat-inactivated fetal bovine serum (Life Technologies) and 1% (vol/vol) penicillin-streptomycin (Sigma) at 37°C and 5% CO_2_.

### Fungal cell culture.

*C. albicans* strains were cultured in YPD medium (1% [wt/vol] yeast extract [Lab M], 2% [wt/vol] peptone [Melford], 2% [wt/vol] dextrose [BDH]). Solid YPD medium was produced by inclusion of 1.5% (wt/vol) agar (Melford). Transformed strains were cultured on synthetic defined (SD) medium (2% [wt/vol] dextrose [BDH], 0.67% [wt/vol] yeast nitrogen base without amino acids [Difco], 1.5% [wt/vol] agar [Melford]). Hyphal growth was induced by culturing fungal strains in RPMI 1640 medium (Life Technologies) for 3 h at 37°C and 5% CO_2_.

### Infection of epithelial cells with *C. albicans.*

Prior to infection, confluent TR146 epithelial cells were serum starved overnight, and all experiments were carried out in serum-free DMEM–F-12 medium. For Western blotting, epithelial cells were infected with *C. albicans* strains at a multiplicity of infection (MOI) of 10 for 2 h. For cytokine and damage assays, cells were infected at an MOI of 0.01 for 24 h. For c-Fos DNA binding assays, cells were infected at an MOI of 10 for 3 h. Following infection, cells were cultured at 37°C and 5% CO_2_.

### Construction of alanine substitution mutants in *C. albicans* Ece1p.

The plasmid CIp10-*ECE1* (3, 32), containing the *ECE1* gene and its upstream and downstream intergenic regions, was used as a parental template for site-directed mutagenesis. Site-directed mutagenesis was performed using the QuikChange site-directed mutagenesis system (Agilent). Alanine substitutions in the *ECE1* gene were screened by restriction endonuclease digestion, and mutations were confirmed by DNA sequencing. Mutagenized constructs were linearized by digestion with StuI and concentrated by ethanol precipitation prior to transformation.

### Transformation of *C. albicans.*

A uridine auxotrophic *ece1* null mutant [*ece1*Δ/Δ (*ura*^−^)] was transformed with 15 µg of linearized construct using a lithium acetate method modified from reference 33. Transformants were selected on SD agar medium and were restreaked onto fresh SD agar three times to ensure stability, and genomic DNA was extracted using phenol-chloroform-isoamyl alcohol and glass bead lysis. Successful integration of each construct into the *C. albicans* genome (at the *RPS1* locus) was confirmed by PCR amplification across the 5′ and 3′ integration sites.

### Construction of *KEX1* reintegrant.

For complementation of the *kex1*Δ/Δ mutant (3), the *KEX1* gene plus upstream and downstream intergenic regions was amplified with primers *KEX1*-comp-F (including a SalI restriction site) and *KEX1*-comp-R (including a ClaI restriction site) and cloned into SalI- and ClaI-digested plasmid CIp10 (32), yielding CIp10-*KEX1*. After linearization with StuI, this plasmid was transformed into a uridine auxotrophic *kex1*Δ/Δ strain, yielding the *kex1*Δ/Δ+*KEX1* complemented strain.

### Preparation of protein extracts.

Epithelial cells were lysed using a modified RIPA buffer (50 mM Tris-HCl, pH 7.4, 150 mM NaCl, 1 mM EDTA, 1% Triton X-100, 1% sodium deoxycholate, 0.1% SDS) containing protease (Sigma-Aldrich) and phosphatase (Perbio Science) inhibitors. Crude lysates were cleared by centrifugation at 4°C, and protein concentration was estimated by bicinchoninic acid (BCA) assay (Thermo Scientific) according to the manufacturer’s instructions.

### SDS-PAGE and Western blotting.

Proteins were resolved by electrophoresis on 12% SDS-PAGE gels using a mini-Protean Tetra cell system (Bio-Rad). Electrophoresed proteins were transferred to a nitrocellulose membrane (Bio-Rad) using a mini-Transblot electrophoretic transfer cell (Bio-Rad). Membranes were blocked in 1× Tris-buffered saline (TBS; Severn Biotech) containing 0.001% (vol/vol) Tween 20 (Acros Organics) and 5% (wt/vol) fat-free milk powder (Sainsbury’s). Primary antibodies diluted (1:1,000) in TBS-Tween and 5% milk (c-Fos) or TBS-Tween and 5% bovine serum albumin (p-DUSP1/MKP1) were added, and membranes were incubated overnight at 4°C with gentle shaking. Following incubation, membranes were washed with 1× TBS containing 0.001% (vol/vol) Tween 20, diluted (1:10,000) horseradish peroxidase (HRP)-conjugated secondary antibody was added, and membranes were incubated for 1 h at room temperature. Membranes were washed as described above and exposed to Immobilon Western chemiluminescent HRP substrate (Millipore) prior to visualization by exposure to film (GE Healthcare). Alpha-actin was used as a loading control.

### Epithelial cell damage assay.

Damage to epithelial monolayers was determined by quantification of lactate dehydrogenase activity using a CytoTox 96 nonradioactive cytotoxicity assay (Promega) according to the manufacturer’s instructions. Porcine lactate dehydrogenase (Sigma) was used to create the standard curve.

### Quantification of secreted cytokines.

Exhausted cell culture medium was collected, and the concentration of cytokines was determined using magnetic microparticles (R&D Systems) specific for human IL-1α, IL-1β, IL-6, G-CSF, and GM-CSF (catalog numbers LUHM200, LUHM201, LUHM206, LUHM214, and LUHM215, respectively) in conjunction with a magnetic Luminex performance assay (Bio-Techne; catalog number LUHM000) and Bio-Plex 200 System (Bio-Rad) according to the manufacturer’s instructions.

### Transcription factor (c-Fos) DNA binding assay.

Epithelial cells were differentially lysed to recover nuclear proteins using a nuclear protein extraction kit (Active Motif) according to the manufacturer’s instructions, and 5 μg of nuclear extract was quantified for c-Fos DNA binding activity using a TransAM DNA binding assay (Active Motif) according to the manufacturer’s instructions.

### Growth curve analysis.

*C. albicans* mutant and control strains were cultured in YPD liquid medium overnight at 30°C in a shaking incubator (180 rpm). Cultured cells were washed twice in sterile phosphate-buffered saline (PBS), and absorbance (600 nm) was adjusted to 0.1 in YPD liquid medium, using a Biochrom WPA CO8000 cell density meter. For each analysis, 200 μl of adjusted culture was added to three individual wells of a 96-well plate. The plate was sealed and maintained at 30°C, and the absorbance (600 nm) was determined every 30 min using a Tecan Infinite 200 Pro plate reader (Tecan Instruments).

### Hyphal length analysis.

*C. albicans* mutant and control strains were cultured in YPD liquid medium overnight at 30°C in a shaking incubator (180 rpm). Cultured cells were washed twice in sterile PBS and adjusted to a concentration of 5 × 10^4^ ml^−1^ in RPMI 1640 medium. For each analysis, 1 ml of adjusted culture was added to 2 individual wells of a 24-well plate, each containing a glass coverslip. The plate was incubated at 37°C, 5% CO_2_, for 3 h, and after incubation, the wells were washed once with PBS and fungal cells were fixed in 4% paraformaldehyde, washed again, and stained with calcofluor white. Coverslips were mounted on microscopy slides, and images were taken of at least 50 fungal cells using fluorescence microscopy. Hyphal length was measured using the software ImageJ (34). Hypha branches were included in the measurement, and nonfilamentous cells were assigned a hyphal length equal to 0.

### Inhibition of secreted aspartic proteinase activity.

*C. albicans* BWP17+CIp30 was cultured under hypha-inducing conditions in the presence of 50 µM pepstatin A (Sigma) to inhibit the activity of Saps. A control culture treated with an equivalent volume of ethanol (vehicle) was prepared in parallel.

### LC-MS/MS analysis of hypha-secreted Ece1p peptides.

Analysis of hypha-secreted Ece1p peptides was optimized for the detection of candidalysin and performed as previously described (3). The methodology used is biased toward the detection of small peptides. Briefly, *Candida* strains were cultured for 18 h under strong hypha-inducing conditions {yeast nitrogen base [YNB] medium containing 2% sucrose, 75 mM MOPSO [3-(*N*-morpholino)-2-hydroxypropanesulfonic acid] buffer, pH 7.2, 5 mM *N*-acetyl-d-glucosamine, 37°C}. Peptides secreted into the exhausted culture medium were enriched by solid-phase extraction (SPE), passed through a 10-kDa-molecular-mass cutoff filter, and resolubilized in 0.2% formic acid in 71:27:2 (vol/vol/vol) acetonitrile (ACN)-H_2_O-dimethyl sulfoxide (DMSO). LC-MS/MS analysis was performed using an Ultimate 3000 nano-LC coupled to a Q Exactive Plus mass spectrometer (Thermo). Peptides were separated on an Accucore C_4_ column (15 cm by 75 µm, 2.6 µm) with a 60 min LC gradient of (A) 0.2% HCOOH in 95:5 H_2_O-DMSO and (B) 0.2% HCOOH in 85:10:5 ACN-H_2_O-DMSO: 0 to 1.5 min at 60% B, 35 to 45 min at 96% B, and 45.1 to 60 min at 60% B. The top 10 precursor ions (full scan at *m/z* 300 to 1,600, *R* = 70k, full width at half maximum [FWHM]) per scan cycle underwent HCD (high energy collisional dissociation) fragmentation (30 V). Resulting MS/MS spectra were monitored at *R* = 17.5k (FWHM). Proteome Discoverer 1.4 (Thermo) and the Sequest HT algorithm were used for protein database searching against *C. albicans* SC5314 (Candida Genome Database [http://www.candidagenome.org]). Mass spectra were searched for both unspecific cleavages (no enzyme) and tryptic peptides up to 4 missed cleavages. The precursor mass tolerance was 10 ppm, and the fragment mass tolerance was 0.02 Da. At least two unique peptides per protein, a false discovery rate of <1%, and cross-correlation (Xcorr) validation (from 2.0 at *z* = 2 up to 3.0 at *z* = 6) were required for positive protein hits.

### RNA extraction from fungi.

*C. albicans* mutant and control strains were cultured in YPD liquid medium overnight at 30°C in a shaking incubator (180 rpm). Cells were washed twice in PBS, and the concentration was adjusted to 1 × 10^7^ cells ml^−1^ in 25 ml RPMI 1640 (hypha inducing) or 5 ml YPD (yeast). For hyphal samples, fungal suspensions were distributed in 150-cm^2^ petri dishes and incubated at 37°C and 5% CO_2_ for 3 h. After incubation, medium and nonadherent *Candida* cells were discarded. Adherent *Candida* cells were rinsed once with ice-cold PBS, loosened with a cell scraper, and collected. For yeast samples, fungal suspensions were cultured at 30°C for 3 h in a shaking incubator (180 rpm). After incubation, cells were collected by centrifugation (3,000 × *g* for 2 min at 4°C) and resuspended in 10 ml ice-cold PBS. Hyphae and yeast samples were washed again with 1 ml ice-cold PBS and centrifuged (3,000 × *g*, 2 min, 4°C), the supernatant was removed, and cell pellets were snap-frozen in liquid nitrogen. Frozen *Candida* pellets were thawed in 600 µl RLT buffer (Qiagen) containing 1% β-mercaptoethanol, mixed with 300 µl acid-washed glass beads (diameter, 0.5 mm), and bead beaten twice at 5,500 rpm for 15 s. Lysates were centrifuged for 2 min at 20,000 × *g*, 4°C, the supernatant was mixed with an equal volume of 70% ethanol (prepared in diethyl pyrocarbonate [DEPC]-water), and total RNA was isolated using the RNeasy minikit (Qiagen) according to the manufacturer’s instructions. RNA integrity and concentration were confirmed using a Bioanalyzer (Agilent).

### Quantification of *C. albicans ECE1* gene expression.

RNA (500 ng) was treated with DNase (Epicentre), and cDNA was synthesized using Superscript III reverse transcriptase (Invitrogen). cDNA samples were used for qPCR with EvaGreen mix (Bio&Sell). Primers (ACT1-F and ACT1-R for *ACT1* and ECE1-F2 and ECE1-R for *ECE1* [Table S4]) were used at a final concentration of 500 nM. qPCR amplifications were performed using a CFX96 thermocycler (Bio-Rad). *ECE1* expression was calculated using the threshold cycle (ΔΔ*C*_*T*_) method, with *ACT1* as the reference gene and *C. albicans* reference strain SC5314 (yeast morphology) as the control sample.

### Nonimmunosuppressed model of OPC infection.

BALB/c mice were purchased from Harlan and housed at King’s College London. A murine model of oropharyngeal candidiasis (OPC) (35) was modified to investigate early-phase gene expression responses to selected *C. albicans* alanine substitution mutants and controls. Briefly, nonimmunosuppressed female BALB/c mice (6 to 8 weeks old, 22 to 25 g) were sedated for 75 min with an intraperitoneal injection of 110 mg/kg of body weight ketamine and 8 mg/kg xylazine, and a swab soaked in sterile saline (vehicle) or 1 × 10^7^ CFU ml^−1^ of *C. albicans* yeast culture (WT, *ece1*Δ/Δ, *ece1*Δ/Δ+*ECE1*, *ece1*Δ/Δ+*ECE1*_Δ184–279_, R31A, R61A, R93A, R61 + R93A, and ALL KA) in sterile saline was placed sublingually for 75 min. After 24 h, mice were sacrificed, the tongue was excised, and RNA was extracted as described below.

### RNA extraction from murine tissue.

Murine tongue tissue was homogenized in RLT lysis buffer (Qiagen) containing 1% β-mercaptoethanol using a gentleMACs dissociator (Miltenyi Biotec), and RNA was extracted using an RNeasy Plus minikit (Qiagen) according to the manufacturer’s instructions.

### Quantification of gene expression from murine tissue.

RNA (600 ng) was treated with Turbo DNase (Invitrogen), and cDNA was synthesized using Superscript IV reverse transcriptase (Invitrogen). cDNA samples were used for qPCR with FIREpol EvaGreen qPCR Mix Plus (ROX) (Solis BioDyne). Primers (complementary to murine beta-actin, *Ccl20*, *Il1b*, *Il6*, and *Csf3* [Table S4]) were used at a final concentration of 400 nM. qPCR amplifications were performed using a RotorGene qPCR system (Corbett). Gene expression was calculated using the two-standard-curve method with murine beta-actin as the reference gene.

### Murine model of oropharyngeal candidiasis.

BALB/c mice were purchased from The Jackson Laboratory and housed at the University of Pittsburgh. Mice were injected subcutaneously with 225 mg/kg cortisone acetate (prepared in 0.05% Tween 20-PBS solution) 1 day before infection. The OPC experiment was performed the following day. Mice were sedated with an intraperitoneal injection of ketamine-xylazine solution (15 mg ml^−1^ ketamine, 1.5 mg ml^−1^ xylazine prepared in sterile saline). A 2.5 mg cotton ball was soaked in *C. albicans* solution (1 × 10^7^ CFU ml^−1^ in sterile PBS) and placed sublingually for 75 min as described in reference 36. Oral swabs were obtained before every experiment to verify the absence of commensal fungi. Mice were sacrificed, the tongue was excised, tissue homogenates were prepared on a gentleMACS dissociator (Miltenyi Biotec), and CFU were determined by plating serial dilutions on YPD agar supplemented with 50 μg ml^−1^ ampicillin.

### Statistical analysis.

All data were analyzed by one-way analysis of variance (ANOVA) with a *post hoc* Dunnett comparison test. In all cases, *P* ≤ 0.05 was taken to be significant. Where data are expressed as “fold change versus vehicle control,” a log transformation was performed prior to performing statistical analysis, to ensure a normal distribution of data.

### Ethics statement.

Murine infections were performed under UK Home Office project license PPL 70/7598 in dedicated animal facilities at King’s College London and under U.S. license 14125154 (with modification number IM-14125154-21). All protocols were approved by the King’s College London ethical review board and the University of Pittsburgh IACUC. Power analysis was used to predetermine sample size. No method of randomization was used to allocate animals to experimental groups. Mice in the same cage were part of the same treatment. The investigators were not blind during outcome assessment.
