# Supplementary figures and images for: Processing of Candida albicans Ece1p Is Critical for Candidalysin Maturation and Fungal Virulence
Source: mBio. 2018 Jan 23;9(1):e02178-17. doi: 10.1128/mBio.02178-17 (PMC5784256; doi:10.1128/mBio.02178-17)

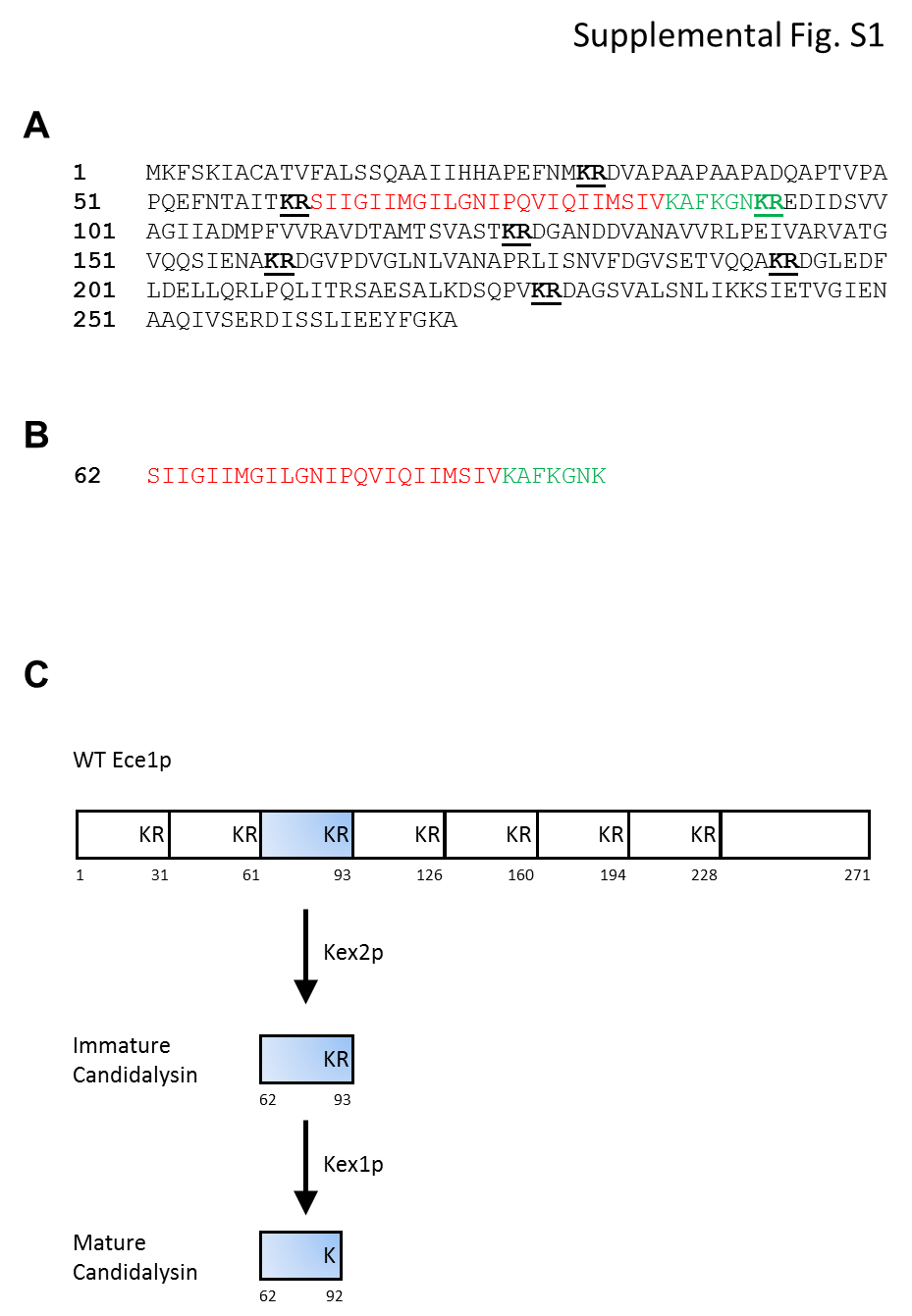

Supplement: FIG S1 [file mbo001183688sf1.tif]

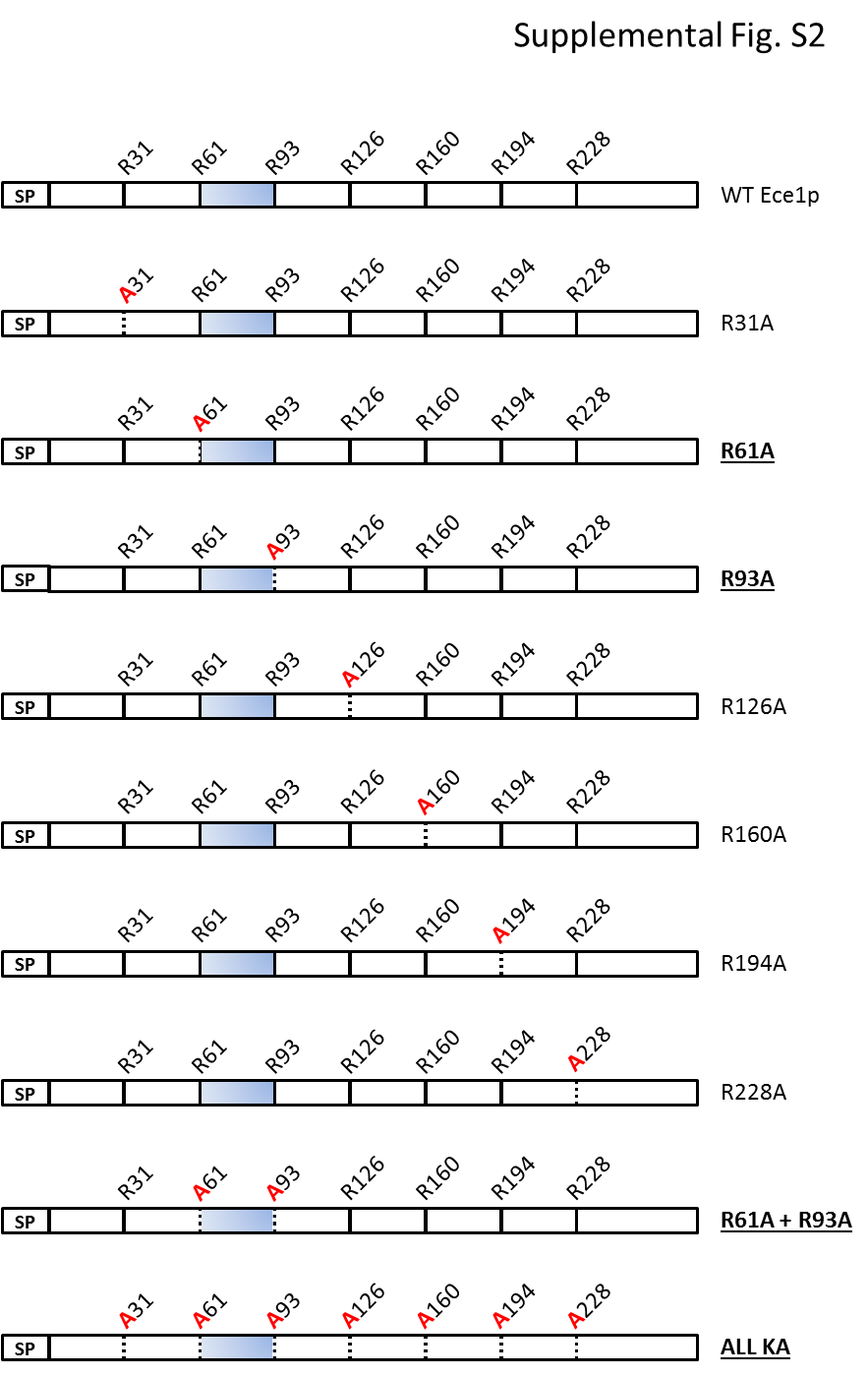

Supplement: FIG S2 [file mbo001183688sf2.tif]

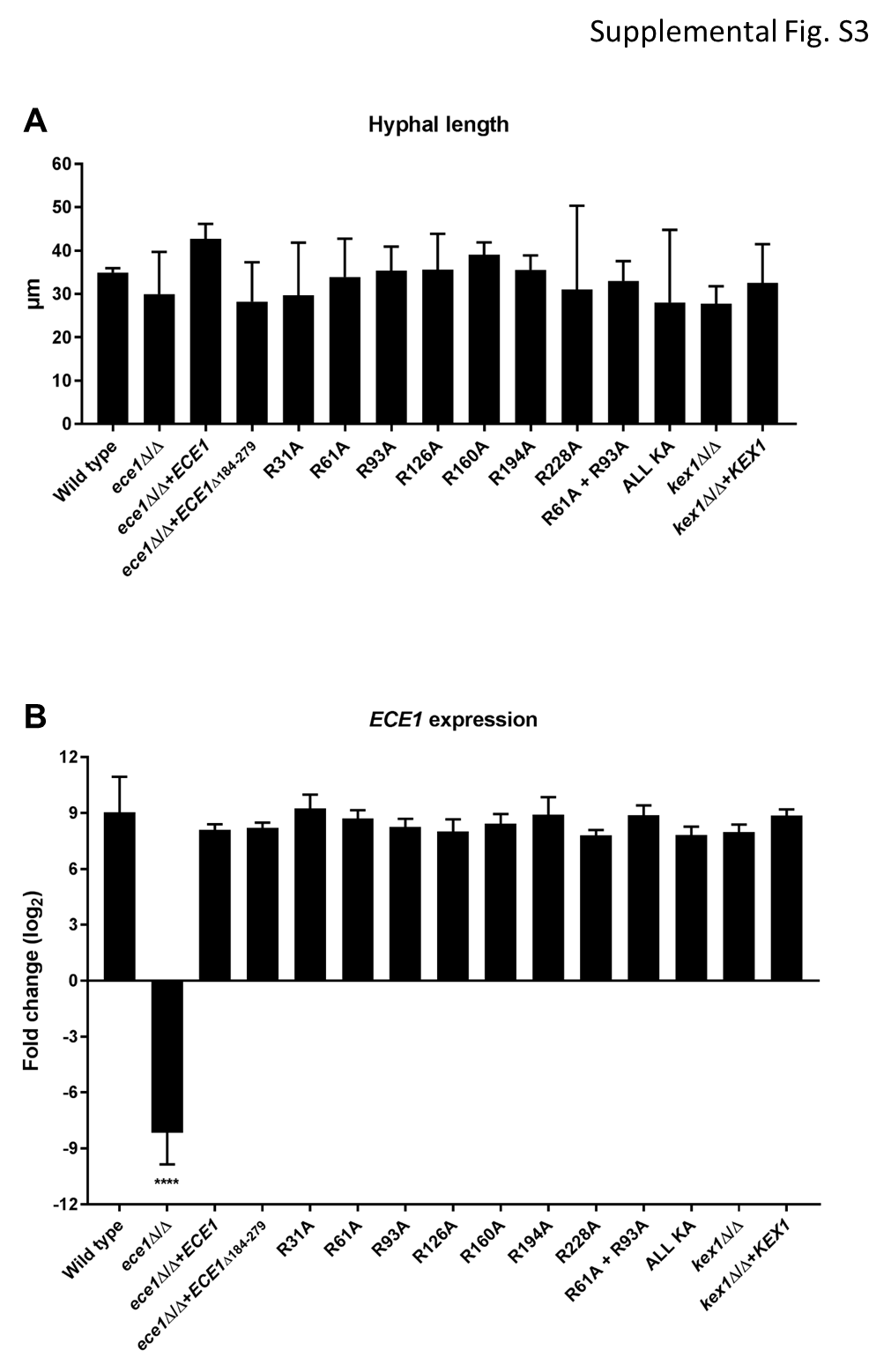

Supplement: FIG S3 [file mbo001183688sf3.tif]

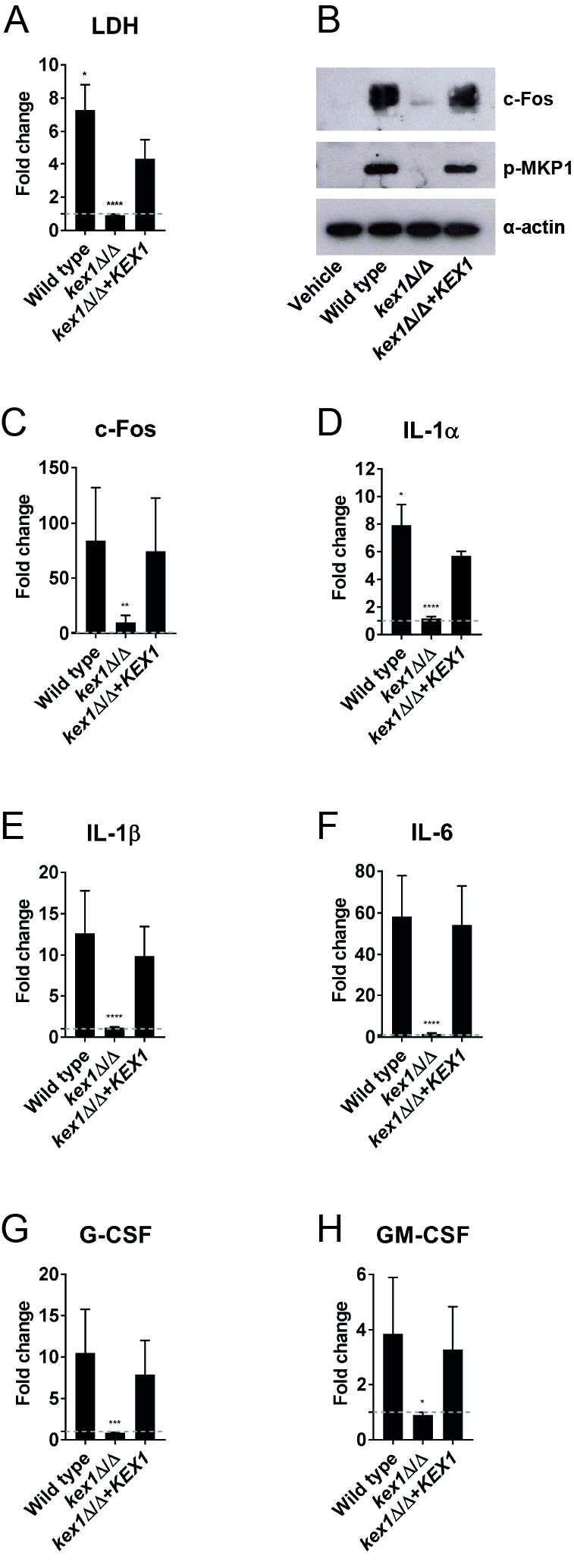

Supplement: FIG S4 [file mbo001183688sf4.tif]
