# Supplementary material for: Processing of Candida albicans Ece1p Is Critical for Candidalysin Maturation and Fungal Virulence
Source: mBio. 2018 Jan 23;9(1):e02178-17. doi: 10.1128/mBio.02178-17 (PMC5784256; doi:10.1128/mBio.02178-17)
Supplement: TABLE S1 [file mbo001183688st1.docx]

**Supplemental Table S1.** Nomenclature and amino acid sequence of Ece1 peptides.

| **Peptide nomenclature** | **Amino acid sequence** |
| --- | --- |
| *Ece1-I* | MKFSKIACATVFALSSQAAIIHHAPEFNM**KR** |
| *Ece1-II* | DVAPAAPAAPADQAPTVPAPQEFNTAIT**KR** |
| P2-P3_DK-61 | DVAPAAPAAPADQAPTVPAPQEFNTAITKA*SIIGIIMGILGNIPQVIQIIMSIVKAFKGNK* |
| *Ece1-III* | SIIGIIMGILGNIPQVIQIIMSIVKAFKGN**KR** |
| Candidalysin | SIIGIIMGILGNIPQVIQIIMSIVKAFKGNK |
| *Ece1-IV* | EDIDSVVAGIIADMPFVVRAVDTAMTSVAST**KR** |
| *Ece1-V* | DGANDDVANAVVRLPEIVARVATGVQQSIENA**KR** |
| P5_DA-19 | DGANDDVANAVVRLPEIVA |
| *Ece1-VI* | DGVPDVGLNLVANAPRLISNVFDGVSETVQQA**KR** |
| *Ece1-VII* | DGLEDFLDELLQRLPQLITRSAESALKDSQPV**KR** |
| P7_DT-19 | DGLEDFLDELLQRLPQLIT |
| P7_DR-20 | DGLEDFLDELLQRLPQLITR |
| P7_SV-12 | SAESALKDSQPV |
| *Ece1-VIII* | DAGSVALSNLIKKSIETVGIENAAQIVSERDISSLIEEYFGKA |
| P8_DK-13 | DAGSVALSNLIKK |
| P8_DA-13 | DISSLIEEYFGKA |

Amino acid sequences of peptides resulting from a hypothetical digestion of Ece1p by Kex2p are designated Ece1-I to –VIII (red, italicised), and their respective KR cleavage sites are highlighted in bold and underlined. The amino acid sequence of Candidalysin (when detected as part of a secreted fusion peptide), is italicised.

All secreted peptides detected by LC-MS/MS are named after the peptide they reside within following Kex2p digestion. The first and last amino acid residue and sequence length are provided.

For example, the seventh peptide sequence derived from Kex2p digestion of Ece1p (*Ece1-VII*) has the amino acid sequence: DGLEDFLDELLQRLPQLITRSAESALKDSQPV**KR**. Peptide “P7_DT-19” is thus a secreted peptide fragment that resides within the *Ece1-VII* sequence that begins with a D, terminates with a T and consists of 19 amino acid residues.

Full details of LC-MS/MS datasets and sequence alignments are provided in Supplemental Dataset S1 and Supplemental Figure S4.
