# Supplementary material for: Processing of Candida albicans Ece1p Is Critical for Candidalysin Maturation and Fungal Virulence
Source: mBio. 2018 Jan 23;9(1):e02178-17. doi: 10.1128/mBio.02178-17 (PMC5784256; doi:10.1128/mBio.02178-17)
Supplement: TABLE S2 [file mbo001183688st2.docx]

**Supplemental Table S2.** LC-MS/MS analysis of *kex1*Δ/Δ hypha-secreted Ece1p peptides; the role of Kex1p in Candidalysin maturation.

| **Strain** | **Experimental Replicate** | **Immature Candidalysin^a^ (PSMs)** | **Mature Candidalysin^b^ (PSMs)** |
| --- | --- | --- | --- |
| BWP17 | 1 | 28 | 715 |
|  | 2 | 26 | 730 |
|  | 3 | 0 | 79 |
| *kex1*Δ/Δ | 1 | 371 | 80 |
|  | 2 | 291 | 49 |
|  | 3 | 71 | 19 |
| *kex1*Δ/Δ+*KEX1* | 1 | 0 | 110 |

**^a^**Amino acid sequence: SIIGIIMGILGNIPQVIQIIMSIVKAFKGNKR

**^b^**Amino acid sequence: SIIGIIMGILGNIPQVIQIIMSIVKAFKGNK

PSM values are semi-quantitative

Full details of LC-MS/MS datasets and sequence alignments are provided in Supplemental Dataset S1 and Supplemental Figure S4.
