# Supplementary material for: Processing of Candida albicans Ece1p Is Critical for Candidalysin Maturation and Fungal Virulence
Source: mBio. 2018 Jan 23;9(1):e02178-17. doi: 10.1128/mBio.02178-17 (PMC5784256; doi:10.1128/mBio.02178-17)
Supplement: TABLE S3 [file mbo001183688st3.docx]

**Supporting Table S3.** *Candida albicans* strains used in this study.

| **Strain name** | **Parent strain** | **Relevant genotype** | **Reference** |
| --- | --- | --- | --- |
| SC5314 (Reference strain – ATCC MYA-2876) |  | Wild type | (13) |
| BWP17 | SC5314 | *ura3*::*λimm434*/*ura3*::*λimm434*  *iro1*::*λimm434*/*iro1*::*λimm434*  *his1*::*hisG*/*his1*::*hisG*  *arg4*::*hisG*/*arg4*::*hisG* | (32) |
| BWP17+CIp30 (Isogenic Wild Type) | BWP17 | *RPS1*/*rps1*::(*URA3*-*HIS1*-*ARG4*) | (33) |
| *ece1*Δ/Δ (*ura*^-^) | BWP17 | *ece1*::*HIS1*/*ece1*::*ARG4* | (3) |
| *ece1*Δ/Δ | *ece1*Δ/Δ (*ura*^-^) | *RPS1*/*rps1*::*URA3* | (3) |
| *ece1*Δ/Δ+*ECE1* | *ece1*Δ/Δ (*ura*^-^) | *RPS1*/*rps1*::(*URA3*-*ECE1*) | (3) |
| *ece1*Δ/Δ+*ECE1*_Δ184-279_ | *ece1*Δ/Δ (*ura*^-^) | *RPS1*/*rps1*::(*URA3*-*ECE1*_Δ184-279_) | (3) |
| *kex1*Δ/Δ | BWP17 | *kex1*::*HIS1*/*kex1*::*ARG4*  *RPS1*/*rps1*::*URA3* | (3) |
| *kex1*Δ/Δ+*KEX1* | BWP17 | *kex1*::*HIS1*/*kex1*::*ARG4*  *RPS1*/*rps1*::(*URA3*-*KEX1*) | This study |
| R31A | *ece1*Δ/Δ (*ura*^-^) | *RPS1*/*rps1*::(*URA3*-*ECE1*_A91G, G92C_) | This study |
| R61A | *ece1*Δ/Δ (*ura*^-^) | *RPS1*/*rps1*::(*URA3*-*ECE1*_A181G, G182C_) | This study |
| R93A | *ece1*Δ/Δ (*ura*^-^) | *RPS1*/*rps1*::(*URA3*-*ECE1*_A277G, G278C_) | This study |
| R126A | *ece1*Δ/Δ (*ura*^-^) | *RPS1*/*rps1*::(*URA3*-*ECE1*_A376G, G377C_) | This study |
| R160A | *ece1*Δ/Δ (*ura*^-^) | *RPS1*/*rps1*::(*URA3*-*ECE1*_A478G, G479C_) | This study |
| R194A | *ece1*Δ/Δ (*ura*^-^) | *RPS1*/*rps1*::(*URA3*-*ECE1*_A580G, G581C_) | This study |
| R228A | *ece1*Δ/Δ (*ura*^-^) | *RPS1*/*rps1*::(*URA3*-*ECE1*_A682G, G683C_) | This study |
| R61A + R93A | *ece1*Δ/Δ (*ura*^-^) | *RPS1*/*rps1*::(*URA3*-*ECE1*_A181G, G182C, A277G, G278C_) | This study |
| ALL KA | *ece1*Δ/Δ (*ura*^-^) | *RPS1*/*rps1*::(*URA3*-*ECE1*_A91G, G92C, A181G, G182C, A277G, G278C, A376G, G377C, A478G, G479C, A580G, G581C, A682G, G683C_) | This study |
|  |  |  |  |
