# Supplementary material for: Processing of Candida albicans Ece1p Is Critical for Candidalysin Maturation and Fungal Virulence
Source: mBio. 2018 Jan 23;9(1):e02178-17. doi: 10.1128/mBio.02178-17 (PMC5784256; doi:10.1128/mBio.02178-17)
Supplement: TABLE S4 [file mbo001183688st4.docx]

**Supplemental Table S4.** Oligonucleotide primers used in this study.

| **Primer Name** | **Application** | **Sequence (5’-3’)** | **Description** |
| --- | --- | --- | --- |
| *ECE1*-SDM 1 F | SDM | ccagaattcaacatgaaggcagatgttgctccagc | Forward mutagenesis primer for construction of E*CE1_A91G, G92C_* (Ece1p R31A) |
| *ECE1*-SDM 2 R | SDM | gctggagcaacatctgccttcatgttgaattctgg | Reverse mutagenesis primer for construction of E*CE1_A91G, G92C_* (Ece1p R31A) |
| *ECE1*-SDM 3 F | SDM | caatactgctattaccaaagcaagtattattgg | Forward mutagenesis primer for construction of *ECE1_A181G, G182C_* (Ece1p R61A) |
| *ECE1*-SDM 4 R | SDM | ccaataatacttgctttggtaatagcagtattg | Reverse mutagenesis primer for construction of *ECE1_A181G, G182C_* _(_Ece1p R61A) |
| *ECE1*-SDM 5 F | SDM | gctttcaaaggtaacaaggcagaagatattgattc | Forward mutagenesis primer for construction of *ECE1_A277G, G278C_* (Ece1p R93A) |
| *ECE1*-SDM 6 R | SDM | gaatcaatatcttctgccttgttacctttgaaagc | Reverse mutagenesis primer for construction of *ECE1_A277G, G278C_* (Ece1p R93A) |
| *ECE1*-SDM 7 F | SDM | ctgttgcttctaccaaggcagatggagctaatg | Forward mutagenesis primer for construction of *ECE1_A376G, G377C_* (Ece1p R126A) |
| *ECE1*-SDM 8 R | SDM | cattagctccatctgccttggtagaagcaacag | Reverse mutagenesis primer for construction of *ECE1_A376G, G377C_* (Ece1p R126A) |
| *ECE1*-SDM 9 F | SDM | ccatcgaaaatgccaaggcagatggcgttccag | Forward mutagenesis primer for construction of *ECE1_A478G, G479C_* (Ece1p R160A) |
| *ECE1*-SDM 10 R | SDM | ctggaacgccatctgccttggcattttcgatgg | Reverse mutagenesis primer for construction of *ECE1_A478G, G479C_* (Ece1p R160A) |
| *ECE1*-SDM 11 F | SDM | ctgttcaacaagctaaggcagatggtcttgaag | Forward mutagenesis primer for construction of *ECE1_A580G, G581C_* (Ece1p R194A) |
| *ECE1*-SDM 12 R | SDM | cttcaagaccatctgccttagcttgttgaacag | Reverse mutagenesis primer for construction of *ECE1_A580G, G581C_* (Ece1p R194A) |
| *ECE1*-SDM 13 F | SDM | cagtcaaccagttaaagcagatgccggctcag | Forward mutagenesis primer for construction of *ECE1_A682G, G683C_* (Ece1p R228A) |
| *ECE1*-SDM 14 R | SDM | ctgagccggcatctgctttaactggttgactg | Reverse mutagenesis primer for construction of *ECE1_A682G, G683C_* (Ece1p R228A) |
| SDM_Seq_1 F | Sequencing | tagtcgtacttgtcatgc | Anneals to *ECE1* 5’ UTR region upstream of ATG start codon |
| SDM_Seq_2 F | Sequencing | atcatcatgagtattgtc | Anneals within the *ECE1* ORF |
| SDM_Seq_3 F | Sequencing | gatggcgtcctggaaactg | Anneals within the *ECE1* ORF |
| URA-F2 F | PCR | ggagttggattagatgataaaggtgatgg | Anneals to *URA3*. Used to confirm integration of transformed inserts in conjunction with RPF-1 in *C. albicans* |
| RPF-1 R | PCR | gagcagtgtacacacacacatcttg | Anneals to flanking (genomic) region of RP10 locus. Used to confirm integration at the *URA3* end of transformed inserts |
| RPF-2 F | PCR | cgccaaagagtttcccctattatc | Anneals to flanking (genomic) region of RP10 locus. Used to confirm integration at the *ECE1* end of transformed inserts |
| ECE-check1 R | PCR | cacaacagagcttctaac | Anneals to 5’ UTR of *ECE1* (intergenic region). Used to confirm integration at the *ECE1* end of transformed inserts. |
| ECE-check2 R | PCR | gtggatgatggcagcttgag | Anneals to the ORF of *ECE1*. Used to confirm integration at the *ECE1* end of transformed inserts |
| *KEX1*-comp-F^1^ | PCR | GGAATGT**c**GA**c**CCATCCAATGTTTAGTGG | Amplification of the *KEX1* gene plus upstream and downstream intergenic regions |
| *KEX1*-comp-R^2^ | PCR | CGTAT**c**GATTGTTTGAACATGATTATGAGC | Amplification of the *KEX1* gene plus upstream and downstream intergenic regions |
| *ACT1*-F | RT-qPCR | tcagaccagctgatttaggtttg | Quantification of actin gene expression in *C. albicans* |
| *ACT1*-R | RT-qPCR | gtgaacaatggatggaccag | Quantification of actin gene expression in *C. albicans* |
| *ECE1*-F2 | RT-qPCR | cactggtgttcaacaatccat | Quantification of *ECE1* gene expression in *C. albicans* |
| *ECE1*-R | RT-qPCR | agcattttcaataccgacag | Quantification of *ECE1* gene expression in *C. albicans* |
| mBeta actin For | RT-qPCR | ggctgtattcccctccatcg | Quantification of actin gene expression in *M. musculus* |
| mBeta actin Rev | RT-qPCR | ccagttggtaacaatgccatgt | Quantification of actin gene expression in *M. musculus* |
| mCCL20 For | RT-qPCR | gcctctcgtacatacagacgc | Quantification of CCL20 gene expression in *M. musculus* |
| mCCL20 Rev | RT-qPCR | ccagttctgctttggatcagc | Quantification of CCL20 gene expression in *M. musculus* |
| mIL-1 beta For | RT-qPCR | gcaactgttcctgaactcaact | Quantification of IL-1 beta gene expression in *M. musculus* |
| mIL-1 beta Rev | RT-qPCR | atcttttggggtccgtcaact | Quantification of IL-1 beta gene expression in *M. musculus* |
| mCsf3 For | RT-qPCR | atggctcaactttctgcccag | Quantification of Csf3 gene expression in *M. musculus* |
| mCsf3 Rev | RT-qPCR | ctgacagtgaccaggggaac | Quantification of Csf3 gene expression in *M. musculus* |
| mIL-6 For | RT-qPCR | tagtccttcctaccccaatttcc | Quantification of IL-6 gene expression in *M. musculus* |
| mIL-6 Rev | RT-qPCR | ttggtccttagccactccttc | Quantification of IL-6 gene expression in *M. musculus* |

^1^ SalI restriction site is underlined and mismatches against the original sequence are shown in bold and lowercase.

^2^ ClaI restriction site is underlined and mismatches against the original sequence are shown in bold and lowercase.
